# Uncommon concurrent pulmonary infections: *Aspergillus fumigatus* and *Lomentospora prolificans* in an Anti-MDA5 antibody-positive dermatomyositis patient

**DOI:** 10.1016/j.mmcr.2024.100689

**Published:** 2024-12-05

**Authors:** Maaya Fukumura, Ryosuke Hiwa, Satomi Yukawa, Yasuhiro Tsuchido, Hajime Yoshifuji, Akio Morinobu

**Affiliations:** a Department of Rheumatology and Clinical Immunology, Graduate School of Medicine, Kyoto University, Kyoto, Japan; b Department of Infection Control and Prevention, Kyoto University Hospital, Kyoto, Japan

**Keywords:** Anti-MDA5 antibody-positive dermatomyositis, Rapidly progressive interstitial lung disease, *Aspergillus fumigatus*, *Lomentospora prolificans*

## Abstract

A 59-year-old female with anti-MDA5 antibody-positive dermatomyositis was treated with prednisolone, tacrolimus, cyclophosphamide, tofacitinib, and plasma exchange. Five months post-treatment, elevated β-D-glucan levels and a pulmonary shadow on CT were noted. *Aspergillus fumigatus* was identified, leading to voriconazole initiation. A new pulmonary cavity lesion later revealed *Lomentospora prolificans*. Considering voriconazole resistance, terbinafine was added, resulting in clinical improvement. Vigilant infection monitoring is crucial during anti-MDA5 antibody-positive dermatomyositis treatment.

## 1 Introduction

Anti-MDA5 antibody-positive dermatomyositis (DM) is among the idiopathic inflammatory myopathies characterized by the concomitant presence of rapidly progressive interstitial lung disease (RP-ILD), often leading to a fatal outcome [1]. Therefore, early initiation of potent immunosuppressive therapy is crucial for managing this condition. Mortality directly linked to RP-ILD typically occurs within the first six months of onset [2], after which various complications become notable concerns. Infections, notably pulmonary infections, are a significant concern for many patients, with a recognized elevated risk compared to other inflammatory myopathies [3,4]. It is crucial to remain vigilant for the simultaneous occurrence of bacterial, viral, fungal, and mycobacterial infections, aiming for their early detection.

Various imaging patterns are observed in pulmonary infections. Given that some pathogens may require long-term treatment, it is essential to identify the causative microorganisms from specimens such as sputum and bronchoalveolar lavage fluid. Fungal infections, which may not typically occur in immunocompetent hosts, require special attention. While *Aspergillus* is a representative of filamentous fungi, it is known that *Lomentospora prolificans* is another common cause of invasive mold infections in lung transplant recipients [5]. Although *Aspergillus* is often susceptible to voriconazole (VRC), *Lomentospora prolificans* exhibits resistance to numerous antifungal agents, including VRC. Therefore, accurately identifying this fungus as a causative microorganism is crucial for ensuring successful clinical outcomes [6].

In this report, we present a case of a patient diagnosed with anti-MDA5 antibody-positive DM and RP-ILD who concurrently developed pulmonary infections with *Aspergillus fumigatus* and *Lomentospora prolificans*. The patient showed a favorable clinical course with treatment comprising a combination of VRC and terbinafine (TBF).

## 2 Case presentation

**Fig. 1:**
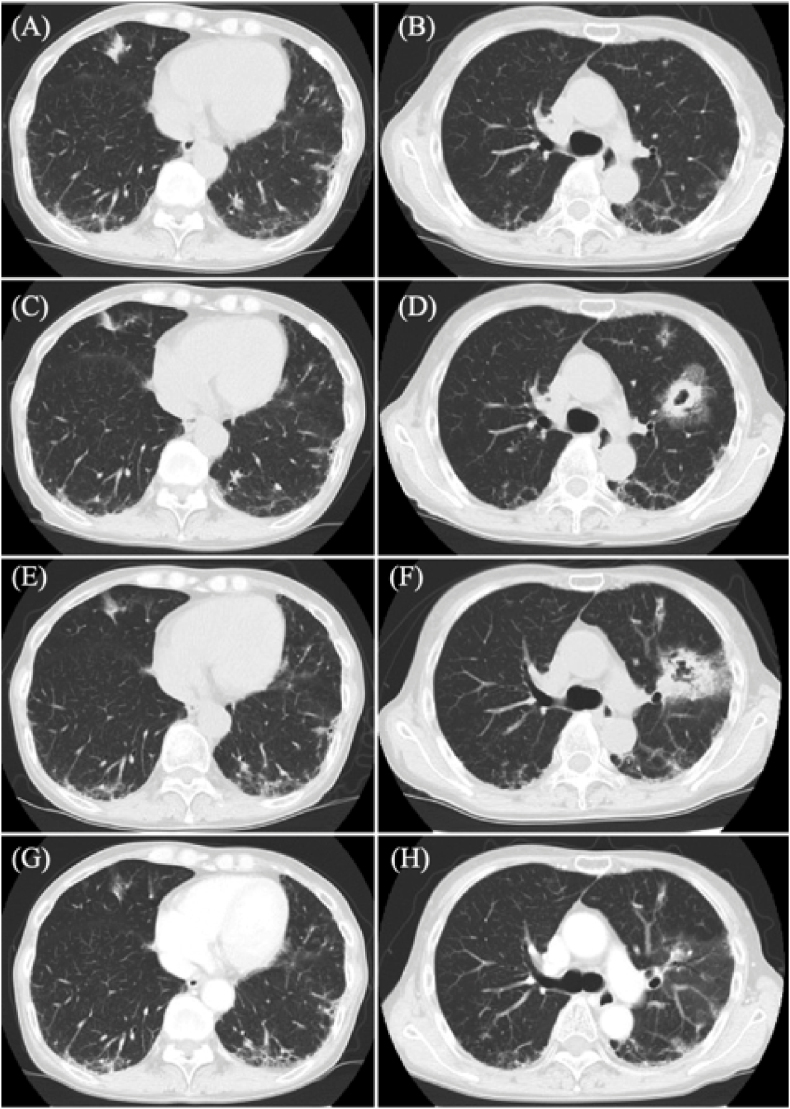
(A–B) Chest computed tomography (CT) revealed a nodular shadow at the base of the right lung five months after the diagnosis of anti-MDA5 antibody-positive dermatomyositis (day 0). (C–D) Three weeks after starting voriconazole (day 22), CT showed a reduction in the size of the known lesion in the right middle lobe, but a new cavitary shadow appeared in the left upper lobe. (E–F) CT after initiating ampicillin-sulbactam and amphotericin B (day 48) showed worsening of the cavitary shadow in the left upper lobe. (G–H) CT one month after the addition of terbinafine (day 65) showed improvement in the cavitary shadow in the left upper lobe.

A 59-year-old female presented to our hospital for investigation of pneumonia and was diagnosed with anti-MDA5 antibody-positive DM and RP-ILD through examinations. Despite treatment with a triple combination therapy (prednisolone, tacrolimus, intravenous cyclophosphamide), interstitial pneumonia worsened. Adding tofacitinib and plasma exchange therapy proved effective, leading to remission. Subsequently, the patient continued receiving intravenous cyclophosphamide every four weeks while gradually tapering prednisolone. Five months after the diagnosis of DM and ILD, the patient was admitted for the eighth course of intravenous cyclophosphamide. Laboratory tests on the day of admission (day 0) showed a high level of β-D-glucan (103.7 pg/ml), and computed tomography (CT) scan showed a new nodular shadow at the base of the right lung [Fig. 1A and B], raising suspicion of fungal infection. Blood *Aspergillus* galactomannan, *Candida* mannan, and *Cryptococcus* glucuronoxylomannan antigens were all negative, and sputum culture tests did not detect any fungi. Bronchoscopy was performed, and *Aspergillus fumigatus* was detected in the culture test of the bronchial lavage fluid specimen. Treatment with VRC 100 mg twice daily was initiated. Following treatment with VRC (day 11), a decrease in β-D-glucan level and improvement in the shadow on chest radiography were confirmed. Considering the suspected exacerbation of ILD based on blood tests and CT scan results, the eighth course of intravenous cyclophosphamide was administered.

However, three weeks after initiating VRC (day 33), the patient presented to the hospital again, complaining of fever. Chest CT scan revealed that the known lesion in the right middle lobe was reduced in size, but a new cavitary shadow appeared in the left upper lobe [Fig. 1C and D]. Laboratory investigations revealed the following: white blood cell count, 5.85 × 10^3^/mm^3^ (88.6 % neutrophils, 2.1 % lymphocytes, and 8.9 % monocytes); hemoglobin level, 10.5 g/dL; platelet count, 25.5 × 10^3^/mm^3^; C-reactive protein level, 1.89 mg/dL; aspartate aminotransferase level, 32 U/L; alanine aminotransferase level, 20 U/L; lactate dehydrogenase level, 397 U/L; γ-glutamyl transpeptidase level, 78 U/L; immunoglobulin G level, 658 mg/dL; ferritin level of 141 ng/mL; Krebs von den Lungen-6 level of 897 U/L, and there were no electrolyte and renal function abnormalities. Although anti-MDA5 antibody titer was 60 index and the β-D-glucan level was 24.0 pg/mL, both levels were lower than the result two weeks earlier. Additional data showed *Aspergillus* galactomannan, *Candida* mannan, and *Cryptococcus* glucuronoxylomannan antigens, interferon-gamma release assay, and anti-*mycobacterium* avium complex antibodies in blood were all negative, and trough concentration of VRC was 1.91 μg/mL. Sputum smear screening for tuberculosis was negative, and the sputum culture test did not reveal causative pathogens such as general bacteria, actinomycetes, or fungi. Bronchoscopy was performed again, but the pathogenic microorganisms could not be identified. Suspecting actinomycosis or VRC-resistant invasive fungal infection, ampicillin-sulbactam was started, and the antifungal agent was changed from VRC to liposomal B (day 36). The cavity shadow worsened after treatment was changed [Fig. 1E and F], and sputum culture tests were submitted repeatedly.

**Fig. 2:**
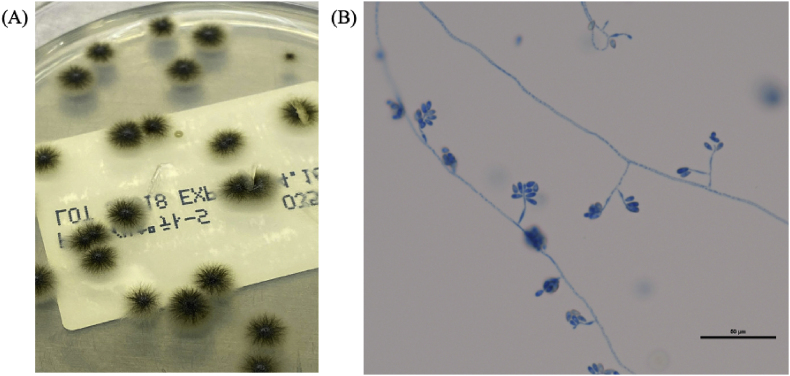
*Lomentospora prolificans* in sputum cultures The sputum cultures revealed the presence of filamentous fungi suspected to be *Lomentospora prolificans*. (A) Macrophotograph of the fungal colony. (B) Micrograph of fungi isolated from the colony.

Eleven days after the switch to liposomal amphotericin B (day 47), multiple sputum cultures revealed the presence of filamentous fungi, with *Lomentospora prolificans* suspected based on morphology and antifungal susceptibility [Fig. 2 and Table 1]. A contrast-enhanced magnetic resonance imaging test of the head revealed no abnormalities. Liposomal amphotericin B was switched back to VRC (day 41) due to persistent fever despite treatment with liposomal amphotericin B. TBF 125 mg a day was added (day 50), assuming VRC-resistant *Lomentospora prolificans*, resulting in a reduction of the cavity shadow on chest CT [Fig. 1G and H]. Based on the clinical course, actinomycosis was ruled out, and we discontinued antibiotics (day 67) while continuing the combination therapy with VRC and TBF. Subsequently, the filamentous fungus was identified as *Lomentospora prolificans* based on β-tubulin gene DNA sequencing analysis (day 73). TBF is scheduled to continue until the cavity shadow on chest CT disappears or becomes fixed.

**Table 1:** Sensitivity results using the micro-broth dilution method (CLSI-M38Ed3) fo*r Aspergillus fumigatus* (A) *and Lomentospora prolificans* (B).

(A)		
Antifungal agents	MIC/MEC	
Micafungin (MCF)	≦ 0.015	μg/mL
Caspofungin (CPF)	0.25	μg/mL
Amphotericin B (AMB)	1	μg/mL
Itraconazole (ITC)	0.5	μg/mL
Voriconazole (VRC)	0.5	μg/mL

(B)		
Antifungal agents	MIC/MEC	
Micafungin (MCF)	16	μg/mL
Caspofungin (CPF)	8	μg/mL
Amphotericin B (AMB)	>16	μg/mL
Itraconazole (ITC)	>8	μg/mL
Voriconazole (VRC)	>8	μg/mL
Terbinafine (TBF)	>64	μg/mL

## 3 Discussion

In the present case, we identified several critical clinical issues. Firstly, interstitial lung disease associated with anti-MDA5 antibody-positive DM requires potent immunosuppressive therapy, highlighting the importance of vigilance for various side effects, including opportunistic fungal infections. Secondly, if new lung laesions develop during antifungal treatment such as VRC, consideration should be given to voriconazole resistant fungal pathogens such as *Lomentospora prolificans*.

Anti-MDA5 antibody-positive DM is recognized for its rapid progression, severity, and poor prognosis. The primary cause of death in the acute phase is RP-ILD, with reports indicating that the underlying disease itself is often fatal within six months of onset [2]. Early and aggressive immunosuppressive treatment is pivotal for a favorable clinical course. The use of triple combination therapy comprising high-dose corticosteroids, calcineurin inhibitors, and cyclophosphamide is established for achieving positive outcomes [7]. However, in cases of disease progression or refractoriness, the efficacy of additional therapeutic modalities such as JAK inhibitors [8] and plasma exchange [9] has been reported. Following the initial treatment phase, complications such as infections become a concern. Notably, pulmonary infections are the most common among all types of infections in non-survivors [4]. Although no cases have been reported in patients with anti-MDA5 antibody-positive dermatomyositis, *Lomentospora prolificans* infections have been documented in patients with connective tissue diseases. For example, a case of osteomyelitis caused by *Lomentospora prolificans* and *Enterobacter cloacae* was treated with meropenem, VRC, and TBF [10]. Additionally, a case of septic arthritis caused by *Lomentospora prolificans* was treated with micafungin and surgical debridement [11].

*Lomentospora prolificans* belongs to the filamentous fungi and is recognized as the second most common cause of invasive mold infections in lung transplant recipients, following *Aspergillus* [5]. Patients with compromised immune status, particularly those experiencing long-term neutropenia, solid organ transplantation, or hereditary/acquired immunodeficiency, are at increased risk for *Lomentospora prolificans* infections [12].

*Lomentospora prolificans* is known for its resistance to many antifungal drugs [6]. European guidelines recommend VRC as the first-line therapy for *Lomentospora prolificans* infections, preferably in combination with surgical debridement. Monotherapy with VRC should be reserved for immunocompetent patients with local infections due to the high mortality rates reported in the central nervous system and disseminated infections [13]. Combination therapy with antifungal agents is considered promising as it enhances therapeutic efficacy at lower concentrations, thus reducing safety concerns, enhancing tolerability, and preventing treatment failure. In a study of 41 patients with invasive *Lomentospora prolificans* infection, combination therapy with VRC and TBF was associated with higher treatment success rates and 28-day survival rates than other antifungal treatment regimens [14]. In our case, the patient developed *Lomentospora prolificans* infection during treatment with VRC. Consequently, we determined that VRC monotherapy was insufficient and added TBF to achieve a synergistic effect. Although resistance to a single drug is observed in in vitro experiments, a synergistic effect can still be expected from a combination of antifungal drugs. In particular, the combination of VRC and TBF has been reported to have the most significant synergistic effect against *Lomentospora prolificans* [15]. Although this patient showed resistance to both VRC and TBF as single agents in in vitro drug susceptibility testing, the clinical condition improved with the combination therapy. The drug susceptibility testing for the combined use of VRC and TBF could not be performed in this case, which represents a limitation. *Lomentospora prolificans* is a rare filamentous fungus and is resistant to VRC in most cases. When there is no improvement with appropriate treatment using antifungal agents to which the fungus shows susceptibility, it is imperative to consider the possibility of concurrent infections with other causative microorganisms. Although rare, a few cases of concurrent fungal infections, including *Lomentospora prolificans*, have been reported. One case involved an orbital abscess caused by *Lomentospora prolificans* and *Aspergillus fumigatus* in a patient with acute myeloid leukemia [16]. Another case described rhino-orbital sinusitis caused by *Lomentospora prolificans* and *Rhizopus oryzae* during treatment with glucocorticoids for COVID-19 infection [17].

We experienced a case of pulmonary infection involving *Lomentospora prolificans* in a patient with anti-MDA5 antibody-positive DM and RP-ILD receiving VRC for *Aspergillus fumigatus* infection. The addition of TBF to VRC resulted in successful outcomes. This case underscores the significance of vigilant infection monitoring during potent immunosuppressive therapy and highlights the need to consider the potential involvement of *Lomentospora prolificans* in such scenarios.

## CRediT authorship contribution statement

**Maaya Fukumura:** Writing – original draft. **Ryosuke Hiwa:** Writing – review & editing. **Satomi Yukawa:** Writing – review & editing. **Yasuhiro Tsuchido:** Writing – review & editing. **Hajime Yoshifuji:** Writing – review & editing. **Akio Morinobu:** Writing – review & editing.
